# Whole-mount optical clearing of rabbit tenuissimus muscle for assessment of muscle spindle morphology

**DOI:** 10.3389/fnins.2026.1858877

**Published:** 2026-08-20

**Authors:** Emily J. Reedich, Brendan C. Moline, Oluwatobi S. Opesade, Cassandra Kramer, Jess E. Glennon, Eden Fraatz, Katharina A. Quinlan, Marin Manuel

**Affiliations:** 1 George and Anne Ryan Institute for Neuroscience, University of Rhode Island, Kingston, RI, United States; 2 Department of Biomedical and Pharmaceutical Sciences, College of Pharmacy, University of Rhode Island, Kingston, RI, United States; 3 Interdisciplinary Neuroscience Program, University of Rhode Island, Kingston, RI, United States

**Keywords:** annulospiral ending, iDISCO, neuromuscular junction, peripheral nervous system, primary afferent, proprioception, α-bungarotoxin

## Abstract

Muscle spindles are essential proprioceptive receptors that provide the sensory foundation for posture, movement coordination, and reflex control. However, their location deep inside muscles and complex three-dimensional architecture have historically limited morphological analysis to labor-intensive techniques such as silver-impregnation, muscle teasing, or serial sectioning followed by volumetric reconstruction. Here, we present a whole-mount optical clearing and multiplex fluorescence imaging workflow that enables *in situ* three-dimensional visualization of muscle spindles in the rabbit tenuissimus muscle, a muscle uniquely rich in spindles. We combined fluorescent labeling of spindle outer capsule cells, sensory, and motor innervation, including intrafusal γ neuromuscular junctions labeled by α-bungarotoxin, with solvent-based optical clearing. We achieved robust and reproducible imaging of the complete spindle structure using both confocal and lightsheet imaging. Notably, we improved on previous methods and were able, for the first time, to label motor endplates with α-bungarotoxin in a way compatible with solvent-based optical clearing, allowing simultaneous visualization of both extrafusal neuromuscular junctions and fusimotor terminals in intact muscles. The rabbit tenuissimus exhibited a remarkably high spindle density (1,600 ± 217 spindles/g), facilitating quantitative morphometric analyses including capsule length, volume, cross-sectional area, and intrafusal fiber numbers. High-resolution volumetric imaging also revealed the complex organization of Ia annulospiral endings within their native anatomical context. This reproducible workflow overcomes major limitations of conventional spindle histology and provides a powerful platform for investigating muscle spindle development, plasticity, aging, and pathology. More broadly, it establishes the rabbit tenuissimus as an exceptional model for studies of proprioceptive organ structure.

## Introduction

Muscle spindles are specialized sensory receptors encapsulated in skeletal muscle that detect changes in muscle length and relay that information to the central nervous system via afferent fibers (Ia and II). They thereby contribute to proprioception and reflex control of muscle tone, forming the afferent limb of the stretch (myotatic) reflex loop (Pierrot-Deseilligny and Burke, 2012). The spindle is composed of intrafusal muscle fibers (two types of nuclear bag and one type of nuclear chain fibers), with contractile polar regions and a noncontractile equatorial region where the sensory endings terminate (Banks, 2015, 2023). The sensitivity of the spindle can be modulated by γ motoneuron (fusimotor) innervation at γ neuromuscular junctions: activation of γ axons (dynamic or static) changes the tension of intrafusal fibers and thus alters the responsiveness of the sensory afferents to stretch (Matthews, 1958; Bishop et al., 1968; Bergenheim et al., 1995; Banks, 2015). Because spindles provide essential feedback for motor control, their structural integrity and proper innervation are critical for normal movement, reflex responses, and adaptation (e.g., motor learning, postural control) (Santuz and Akay, 2023; Bartels and Harrison, 2025).

The tenuissimus muscle in certain species (e.g., cat, rabbit, hamster) has historically been used as a model preparation in spindle physiology and fusimotor studies. The thin, elongated architecture and relative simplicity of this muscle is ideal to study muscle spindles. Their particular arrangement in the muscle allows spindles and sensory and motor axons to be traced more easily than in denser muscles (Butler, 1980). Because the tenuissimus is a relatively thin muscle, light scattering and penetration issues are more manageable in whole-mount imaging. With the advent of tissue clearing techniques [solvent-based, aqueous-based, and hydrogel-based; (Ueda et al., 2020)], this approach can be further refined to offer unprecedented access to the structure of muscle spindles in healthy and disease models.

One hurdle to muscle spindle visualization in optically cleared skeletal muscle is the reported incompatibility between the primary reagent for visualizing γ neuromuscular junctions [fluorescent dye-conjugated α-bungarotoxin (BTX)] and key chemicals involved in clearing (Zhan et al., 2023; Zhu et al., 2026). No previous study has successfully combined α-BTX labeling of γ endplates with iDISCO and solvent-based clearing in skeletal muscle. In this study, we adapt a tissue clearing + immunofluorescence pipeline to the tenuissimus muscle, allowing the visualization of the entire architecture of muscle spindles in three dimensions. Given the central role of spindles in motor control, our approach provides a novel way to study muscle spindle morphology, connectivity, and plasticity in health and disease, *in situ*.

## Methods

### Muscle dissection

#### Ethics statement

All experimental procedures involving animals were approved by the University of Rhode Island’s Institutional Animal Care and Use Committee and performed in accordance with the National Institutes of Health Guide for the Care and Use of Laboratory Animals.

#### Animal housing and husbandry

Neonatal New Zealand White rabbit kits were born to dams bred in-house in our colony that is derived from Charles River Laboratories, Inc. (Wilmington, MA). Rabbits were housed in a controlled vivarium with a 12:12 h light–dark cycle and had access to food and water *ad libitum*.

#### Isolation of the tenuissimus muscle

In this study, we used 21 muscles isolated from 13 rabbits of mixed sex (3 male, 7 female, 3 unknown) from 5 independent clearing preparations. Experiments were conducted between postnatal day (P) 5 and 14, when rabbits were pre-pubertal; sexual maturity is not reached until approximately 6 months of age in this species.

**Figure 1:**
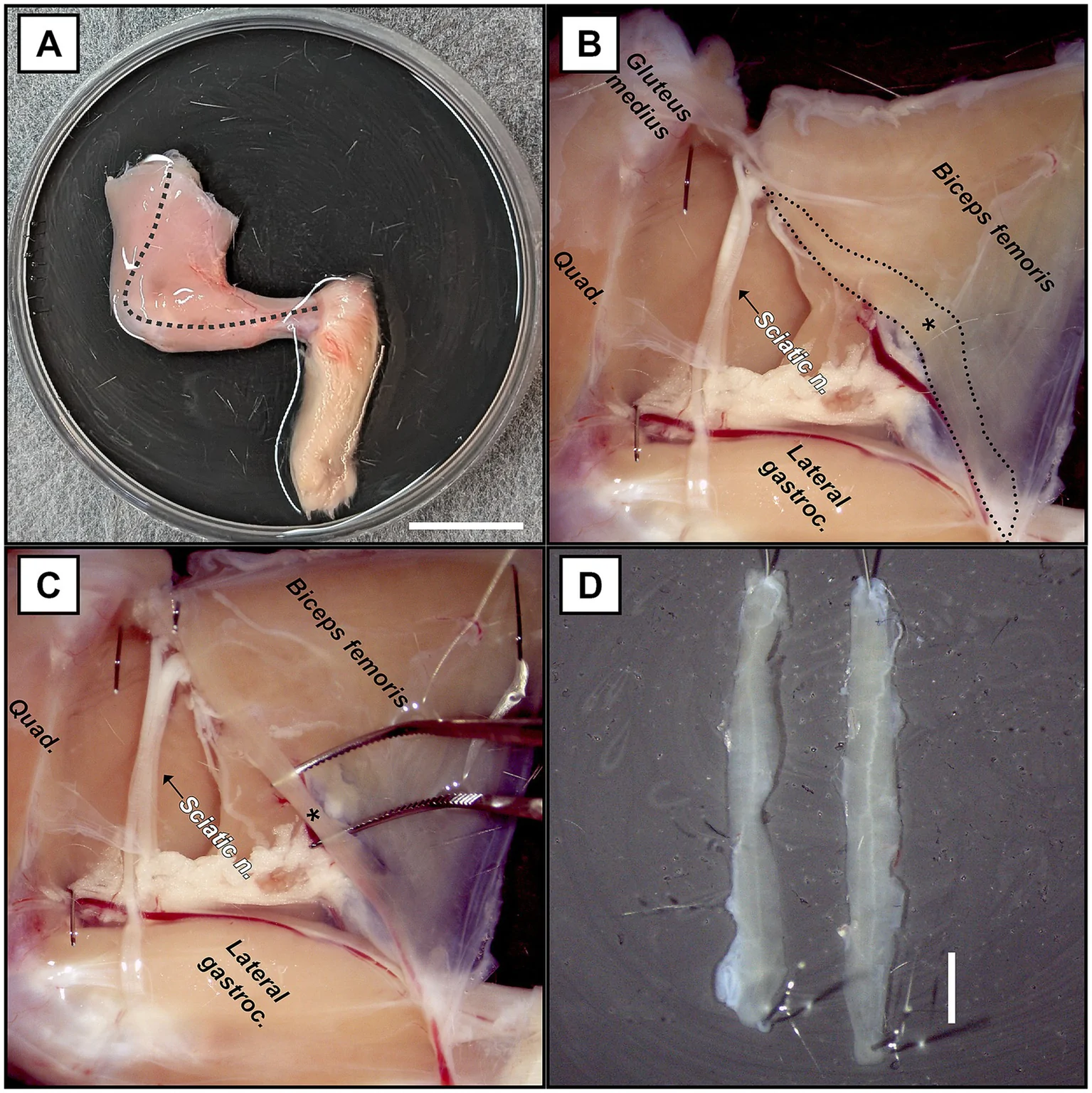
Dissection of the tenuissimus muscle. **(A)** The neonatal rabbit hindlimb is pinned in a lateral recumbent position to a Sylgard-lined petri dish with the knee joint at a 90° angle. The dashed line shows the cut performed to reflect the biceps femoris and expose the tenuissimus muscle. Scale bar: 20 mm. **(B)** The tenuissimus (*) is visible upon medial reflection of the biceps femoris, with which it shares a tendon of insertion along the tibia. **(C)** The tenuissimus (*) is isolated by blunt dissection from the biceps femoris and proximal-to-distal resection. **(D)** Two examples of the isolated tenuissimus; a characteristic neurovascular bundle runs along its midline. Scale bar: 3 mm. Gastroc, gastrocnemius; N, nerve; Quad, quadriceps femoris.

Rabbit kits were euthanized by intraperitoneal injection of Euthasol Euthanasia Solution (pentobarbital sodium and phenytoin sodium; Patterson Veterinary, cat. # 07-805-9296), followed by decapitation with standard surgical scissors (Fine Science Tools, cat. # 14001-018). The fur over the hindlimb was wetted with 70% ethanol (Pharmco, cat. # 111000200) and removed with Iris scissors (Fine Science Tools, cat. # 14060-09) to expose the underlying musculature. The entire hindlimb was dissected from the rest of the carcass with standard surgical scissors (Fine Science Tools, cat. # 14008-14) and pinned to a petri dish (CellTreat, cat. # 229620) lined with Sylgard (Dow, cat. # 01696157) in a lateral recumbent orientation with the knee joint at a 90° angle (Figure 1A). The hindlimb was submerged in chilled phosphate buffered saline (1× PBS). 1× PBS was prepared from a 10× PBS stock (pH 6.8) with the composition: 76.8 mM Na_2_HPO_4_ (Millipore Sigma, cat. # S3264), 26.7 mM Na_2_HPO_4_ . H_2_O (Millipore Sigma, cat. # 71504), and 1.54 M NaCl (Millipore Sigma, cat. # S9888).

An incision was made with spring scissors (Fine Science Tools, cat. # 15651-11) by the patella between the fascia lata and the biceps femoris muscle. The incision was extended medially to the caudal fascia, separating the tensor fasciae latae and gluteus medius muscles from the gluteus maximus. The original incision by the patella was extended through the biceps femoris tendon of insertion and along the tibia distally to the Achilles tendon. The transected biceps femoris tendon of insertion was held with forceps (Fine Science Tools, cat. # 11009-13) and reflected towards the midline to expose the tenuissimus muscle (Figure 1B), which originates at the transverse process of the second caudal vertebra and shares its transected tendon of insertion with the biceps femoris (Chin, 1957; Walker, 1980; Patten and Ovalle, 1992). The tenuissimus was gently blunt dissected along the biceps femoris (Figure 1C) using blunt curved forceps (Fine Science Tools, cat. # 11370-31); next, it was held by its tendon of origin and isolated by resecting it distally to its transected tendon of insertion with small spring scissors (Fine Science Tools, cat. # 91501-09). The isolated tenuissimus was transferred to a weighing dish lined with 1× PBS-dampened gauze and the initial muscle mass was recorded. Lastly, the muscle was photographed on 5 mm graph paper and its initial length was measured using the segmented line tool in Fiji (version 2.14.0/1.54f).

### Optical clearing

#### Labeling intrafusal fiber motor end plates with α-BTX and tissue fixation

The isolated tenuissimus was transferred to a polypropylene microfuge tube containing α-BTX-Alexa Fluor 647 conjugate (Life Technologies, cat. # B35450) diluted 1:250 in 1× PTwH, a solution made of 1× PBS (pH 7.4) with Tween-20 (Millipore Sigma, cat. # P9416; 0.2%) and heparin (Millipore Sigma cat. #H3393; 10 μg/mL) and incubated on a shaker plate at room temperature in the dark for 1 h to fluorescently label motor end plates. All proceeding incubations were also performed in the dark. The muscle was washed once quickly then 4 × 5 min in chilled 1× PBS on a shaker plate at room temperature, and transferred to a Sylgard-lined petri dish. Insect pins (Fine Science Tools, cat. # 26002-20) were used to hold the tenuissimus in a linear position in the dish (Figure 1D); care was taken to ensure the muscle was not stretched. The muscle was then immersion-fixed in 4% paraformaldehyde (Millipore Sigma, cat. #P6148; diluted in 1× PBS; pH 7.4) at 4 °C overnight. The next day, the tissue was washed 3 × 10 min in 1× PBS and stored in 1× PBS with sodium azide (Teknova, cat. # 50209; 0.05%) until immunolabeling. Of note, we tested variations in α-BTX application, and found that increased concentration (1:100), lowered temperature (4 °C for 2 h), and *in vitro* incubation (room temperature for 4 h) in carbogenated physiological saline [formulated as in Reid et al. (1999)] all yielded similar fluorescence intensity of α-BTX labeling.

#### Immunolabeling of spindle annulospiral endings and outer capsule cells

A methanol gradient was used to permeabilize tenuissimus muscles for immunolabeling. We adapted our methanol pre-treatment protocol from (Renier et al., 2014), choosing to omit the hydrogen peroxide-based tissue bleaching step in order to preserve α-BTX-Alexa Fluor 647 fluorescence (Renier et al., 2014). The samples were incubated (all volumes: 1 mL) on a shaker plate at room temperature, for 1 h each, in: 50% methanol (diluted in 1× PBS), 80% methanol (diluted in 1× PBS); then 2 × 1 h in 100% methanol (Fisher Scientific, cat. # A412-4). The sample was next incubated on a shaker plate at 4 °C overnight in 20% dimethyl sulfoxide (DMSO; Millipore Sigma, cat. # 472301; diluted in methanol). The next day, the sample was incubated on a shaker plate at room temperature, for 1 h each, in: 80% methanol (diluted in 1× PBS), 50% methanol (diluted in 1× PBS); followed by 2 × 1 h in 1× PBS then 2 × 1 h in 0.2% Triton X-100 (Millipore Sigma, cat. #T8787).

To visualize spindle innervation, we performed immunolabeling of neurofilament and synaptic vesicle glycoprotein 2, which labels motor axons, their terminals, and sensory axons including Ia annulospiral endings. Outer capsule cells were labeled by immunostaining of glucose transporter 1 (GLUT1), a recently discovered marker of spindle capsules (Bornstein et al., 2023). We adapted our iDISCO protocol from Renier et al. (2014). Immediately after pretreatment, samples were incubated (all volumes: 1 mL) in 0.2% Triton X-100, 20% DMSO, 0.3 M glycine (Millipore Sigma, cat. # G7403) (diluted in 1× PBS) inside an incubator-shaker at 37 °C overnight. The samples were blocked in 0.2% Triton X-100, 10% DMSO, 6% goat serum (Jackson ImmunoResearch, cat. # 005-000-121, lot # 178831) (diluted in 1× PBS) inside an incubator-shaker at 37 °C for 1 day. The samples were washed on a shaker plate at room temperature for 2 × 1 h in PTwH. The samples were incubated in primary antibody cocktail (diluted in 5% DMSO, 3% goat serum in PTwH) inside an incubator-shaker at 37 °C for 2 days. Primary antibody cocktail consisted of mouse anti-synaptic vesicle glycoprotein 2 (DSHB, cat. # SV2-s, lot # 11/7/24; diluted 1:20), mouse anti-neurofilament (DSHB, cat. # 2H3-s, lot # 1/25/24; diluted 1:100), and rabbit anti-GLUT1 (Abcam, cat. # ab115730, lot # 1030236-165; diluted 1:200). The samples were washed on a shaker plate at room temperature for 1 day in PTwH (the buffer was replaced at 1 h, 2 h, and 4 h marks). Next, the samples were incubated in secondary antibody cocktail (diluted in 3% goat serum in PTwH) inside an incubator-shaker at 37 °C for 1 day. Secondary antibody cocktail was composed of Alexa Fluor 555 AffiniPure Goat Anti-Mouse IgG (H+L) (Jackson ImmunoResearch, cat. #115–565-146, lot # 175650; diluted 1:500) and Alexa Fluor 488 AffiniPure Goat Anti-Rabbit IgG (H+L) (Jackson ImmunoResearch, cat. # 111-545-045, lot # 156896; diluted 1:500). Lastly, the samples were washed on a shaker plate at room temperature for 1 day in PTwH (the buffer was replaced at 1 h, 2 h, and 4 h marks). To characterize non-specific fluorescence, negative control samples included those to which α-BTX-647 was not applied, and those to which secondary but not primary antibody cocktail was applied (Supplementary Figure 1).

#### Myonuclei counterstaining

To test whether our protocol was compatible with four-plex fluorescent staining, nuclei were counterstained with a monomeric cyanide nucleic acid stain in a subset of samples. In this case, after the last wash described above, samples were incubated in PO-PRO-1 Iodide (Invitrogen, cat. # P3581, lot # 2911365; 15 μM in PTwH) inside an incubator-shaker at 37 °C overnight. Afterwards, they were washed on a shaker plate at room temperature for 1 day in PTwH (the buffer was replaced at 1 h, 2 h, and 4 h marks).

#### Optical clearing

We adapted FDISCO (Qi et al., 2019) for solvent-based optical clearing of fluorescently labeled tenuissimus muscles. Samples were delipidated and dehydrated by incubation on a shaker plate at 4 °C, for 1.5 h each (all volumes: 1 mL), in: 50% tetrahydrofuran (THF, Millipore Sigma, cat. # 360589), 0.01% triethylamine (Millipore Sigma, cat. # 471283) in ultrapure water; 70% THF, 0.01% triethylamine in ultrapure water; 80% THF, 0.01% triethylamine in ultrapure water. The samples were then incubated in 100% THF, 0.01% triethylamine on a shaker plate at 4 °C for 2 × 2 h. For refractive index matching, the samples were incubated (volume: 1 mL) in 100% dibenzyl ether (DBE; Millipore Sigma, cat. # 33630) at 4 °C for 1 h; this step rendered the samples transparent. The samples were incubated in a fresh aliquot of 100% DBE (volume: 2 mL) at 4 °C overnight. The following day, each cleared muscle was photographed on 5 mm graph paper and its final length was measured using the segmented line tool in Fiji (version 2.14.0/1.54f). Muscle shrinkage was approximated as:


(Initial length−Final length)÷Initial length×100%

The samples were stored in 100% DBE in the dark at 4 °C until microscopy. We note that our samples were also amenable to room temperature storage and imaging in the non-toxic alternative mounting medium, ethyl cinnamate (100%; Millipore Sigma, cat. # 112372).

### Microscopy

#### Epifluorescent and confocal microscopy

For epifluorescent and confocal microscopy, the samples were mounted onto microscope slides (Globe Scientific, Inc., cat. # 1380-20) using the solvent used for refraction index matching (100% DBE or 100% ethyl cinnamate) as the mounting medium. For thicker samples, a thin barrier of vacuum grease (DuPont MOLYKOTE® High Vacuum Grease) can be applied around the coverslip (#1.5; VWR, cat. # 48393-241) to seal in the mounting medium.

Widefield epifluorescent images were acquired on a Nikon ECLIPSE Ti2-E inverted microscope with a motorized stage and a Sola light engine (Lumencor). Observations were performed using a Nikon CFI Plan Apochromat Lambda D 10×/0.45NA air objective, a DAPI Filter Set (Nikon, cat. #96360, excitation: 350/50, dichroic: 400, emission: 460/50) for PO-PRO-1 Iodide, a GFP Filter Set (Nikon, cat. # 96362, excitation: 470/40, dichroic: 495, emission: 525/50) for Alexa Fluor 488, a DsRed/TRITC/Cy3 Filter Set (Nikon, cat. # 96394, excitation: 554/23, dichroic: 573, emission: 609/54) for Alexa Fluor 555, and a Cy5 Filter Set (Nikon, cat. # 96232, excitation: 618/50, dichroic: 652, emission: 698/70) for Alexa Fluor 647. Images were acquired using a Zyla 4.2 sCMOS camera (Andor; 12bit, gain 4, rolling shutter, no binning) and NIS-Elements AR v.6.10.01 (Nikon).

Confocal Z-stacks were acquired on a Nikon ECLIPSE Ti2-E inverted microscope with a motorized stage and a LUN4 laser engine (405/488/561/640 nm, 15 mW), equipped with a Nikon C2+ galvo scanning head, and two C2-DUVB GaAsP detector units. Images were acquired with a CFI Plan Apochromat Lambda D 20×/0.8NA air objective, CFI Plan Apochromat Lambda D 40×/0.95NA air objective, a S Plan Fluor ELWD 40x/0.60NA air objective, or a Plan Apochromat 60×/1.4NA oil immersion objective, optionally with a 1.5× zoom lens inserted in the light path, and NIS-Elements AR v.6.10.01 (Nikon). Details about acquisition parameters are provided in the legends of the relevant figures.

#### Lightsheet microscopy

For lightsheet microscopy, the samples were mounted onto the sample holder with superglue (J-B Weld, SuperWeld) and submerged in the imaging chamber containing the chemical used for refraction index matching (100% DBE or 100% ethyl cinnamate). Lightsheet volumetric images were acquired on either a MegaSPIM (LifeCanvas Technology, Cambridge, MA) or a ZEISS Lightsheet 7 light-sheet fluorescence microscope (Carl Zeiss, Germany). The MegaSPIM illumination and detection are performed using long-working-distance optics positioned at a 45° angle relative to the sample plane. Excitation was provided using a 561 nm laser line. Fluorescence was detected using a 22×/0.7 NA multi-immersion dipping objective. Emitted fluorescence was captured using a Hamamatsu ORCA-Fusion sCMOS camera (2,304 × 2,304 pixels). Raw image stacks were computationally processed using the MegaSPIM post-processing pipeline, including deskewing and stitching, to generate isotropic three-dimensional image volumes suitable for downstream visualization and quantitative analysis. The movie was generated using Arivis Pro v.4.2.1.

The Lightsheet 7 uses two illumination objectives and perpendicular detection optics. Illumination was provided via fused dual light-sheets, with pivot scanning enabled. Images were acquired using a 20×/1.0NA Plan-Neofluar objective, coupled to a PCO.Edge 4.2 sCMOS camera (2,048 × 2,048 px). Laser excitation was performed at 488, 561, and 635 nm; signals were separated using an LBF 405/488/561/640 filter cube; images were acquired in two sequential tracks in continuous Z-stack mode with 10% tile overlap, pivot scan enabled, and track switching via Z-stack multitrack acquisition, implemented through Zen Black 3.0 software.

### Morphological analyses

A subset of optically cleared muscles (unilateral samples from *N* = 8 rabbits from two independent clearing preparations) were designated for spindle density quantification by epifluorescence microscopy at 10× magnification. The number of spindles per muscle was counted using neurofilament, synaptic vesicle glycoprotein 2, and/or GLUT1 immunofluorescence as spindle markers. As described by (Voss, 1937), the spindle density of each sample was calculated as:


Number of spindles÷Initial muscle mass(ing)

#### Morphometry of spindle capsules

A subset of optically cleared muscles (unilateral samples from *N* = 5 rabbits from one independent clearing preparation) were designated for spindle capsule morphometry by lightsheet microscopy and segmentation of GLUT1 immunofluorescence. Lightsheet tiled z-stacks were loaded in Arivis Pro v.4.5.0 and segmented using the built-in pipeline “Detect Big Structures Manual.” The thresholds for segmentation were adjusted manually for each spindle (*N* = 10), and we used a filter with a cutoff size of >100,000 μm^3^ to eliminate all smaller segmented objects. After completion of the pipeline, some cleaning up and manual splitting of objects were required as the pipeline also often segmented the main nerve present in the picture. In particular, the branch innervating the spindle was often segmented together with the capsule and needed to be split off before obtaining measurements.

Once the capsules were segmented, we used the automated measurements of Arivis to obtain (1) the total volume of the spindle, (2) the length of the spindle, as the maximum Feret diameter of the segmented volume, and (3) the maximum cross-sectional area in the XZ plane.

#### Counts of intrafusal fibers

A subset of optically cleared muscles (unilateral samples from *N* = 8 rabbits from three independent clearing preparations) were designated for quantification of intrafusal fibers using confocal photomicrographs. Nine muscle spindles were analyzed and the number of intrafusal muscle fibers in each spindle was carefully assessed using z-stacks and volumetric images. Individual fibers were identified based on the neurofilament and synaptic vesicle glycoprotein 2-labeled annulospiral coils in the equatorial region of the spindle, and on the faint GLUT1 labeling that was visible around each fiber.

#### Signal-to-noise ratio (SNR) estimation

For each fluorescence channel, we quantified the background and signal intensities as a function of depth (Z-axis) from the 3D image stack. At each z-plane, we computed two statistics across the XY dimension. Background intensity was estimated as the median intensity of the image at each depth, which provides a robust measure that is less influenced by sparse bright features. Signal intensity was estimated as the maximum intensity at each depth, capturing the brightest labeled structure present at that z-position. To combine all measurements, we normalized the depth of each image by the total depth of the stack, and because confocal images are acquired at 12 bits and lightsheet images at 16 bits, we also normalized the intensities of the images to the [0–1] range.

## Results

### Solvent-based optical clearing enabled visualization of fluorescently labeled muscle spindles

**Figure 2:**
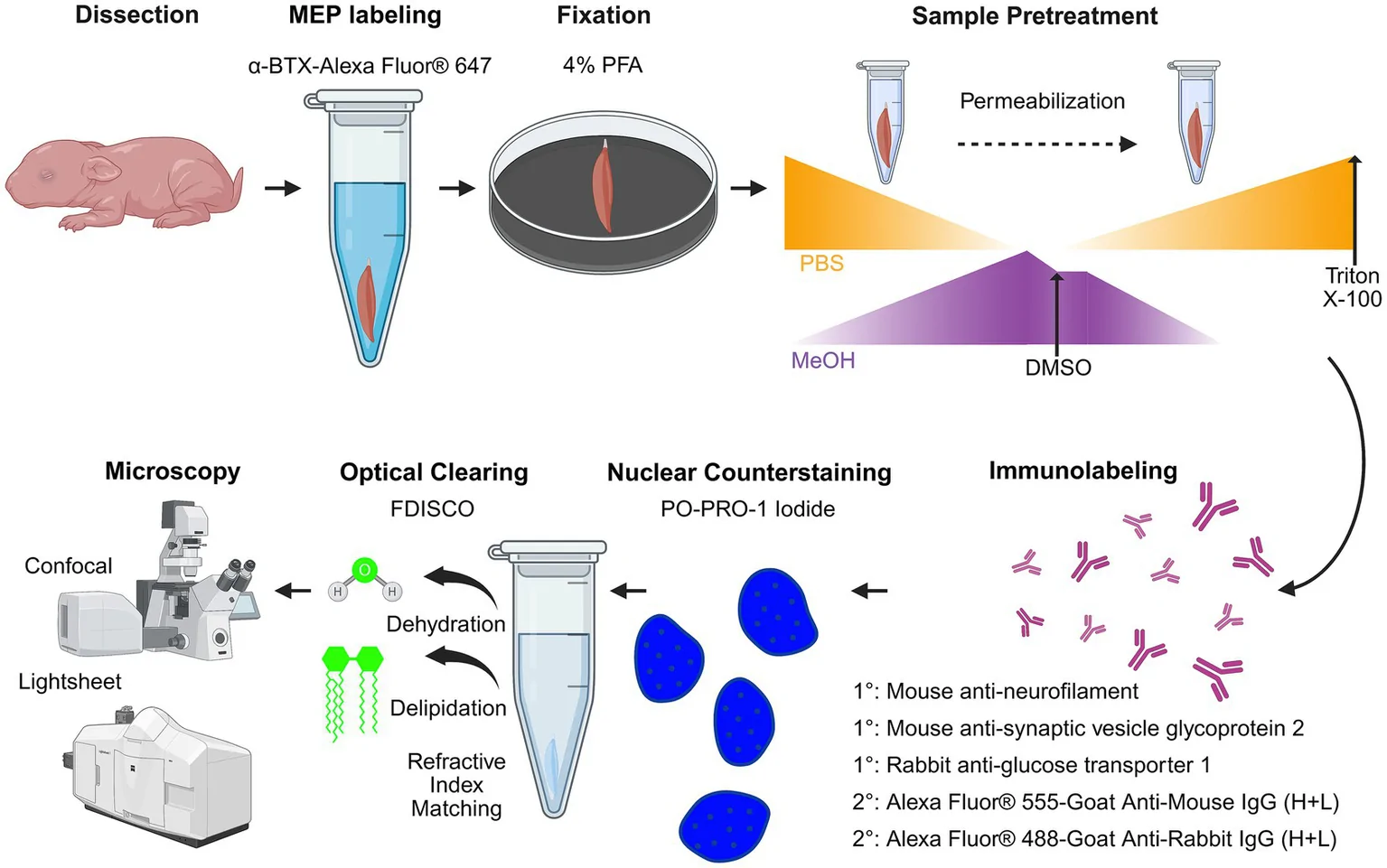
Immunolabeling and optical clearing workflow for three-dimensional visualization of muscle spindles in the tenuissimus. The tenuissimus muscle is dissected from the neonatal rabbit and incubated in α-BTX-Alexa Fluor 647 to label neuromuscular junctions (including fusimotor terminals); then, the sample is pinned flat and chemically fixed by immersion in 4% paraformaldehyde. After fixation, the tenuissimus is permeabilized for immunofluorescence by methanol pretreatment. Antibodies against neurofilament, synaptic vesicle glycoprotein 2, and glucose transporter 1 are used to immunolabel spindle innervation and outer capsule cells, respectively. Nuclei are counterstained with the monomeric cyanide nucleic acid stain PO-PRO-1 Iodide. We adapted the solvent-based optical clearing protocol FDISCO (Qi et al., 2019) to achieve tissue dehydration, delipidation, and refractive index matching of the fluorescently labeled tenuissimus. Muscle spindles in the optically cleared tenuissimus are visualized and volume-imaged using confocal or light-sheet microscopy. Figure made with Biorender. BTX, bungarotoxin; DMSO, dimethyl sulfoxide; IgG, immunoglobulin G; MeOH, methanol; MEP, motor end plate; PBS, phosphate buffered saline; PFA, paraformaldehyde.

The tenuissimus is a long, thin, fusiform muscle that spans across both the knee and the hip in many species. It is thought to play an important role in proprioceptive sensory feedback for the hindlimb (Patten and Ovalle, 1992). In this study, we developed a multiplex fluorescent labeling and optical clearing workflow for three-dimensional visualization of muscle spindles in the rabbit tenuissimus (Figure 2). We labeled spindle poles by staining γ-neuromuscular junctions with fluorescent *α*-BTX and chemically cross-linked this reagent in place by immersion fixation in paraformaldehyde. Then, we adapted the immunofluorescence framework of iDISCO (Renier et al., 2014) to fluorescently label neurofilament and synaptic vesicle glycoprotein 2 for visualization of spindle sensory and motor innervation; spindle capsules were visualized by immunofluorescent labeling of glucose transporter 1 (GLUT1) in outer capsule cells. As proof-of-principle for four-plex fluorescent staining, myonuclei were counterstained with the monomeric cyanide nucleic acid stain PO-PRO-1 Iodide. Finally, we adapted the solvent-based optical clearing pipeline of FDISCO (Qi et al., 2019) to achieve dehydration, delipidation, and refractive index matching of the fluorescently labeled tenuissimus. This workflow enabled volume imaging of the optically cleared tenuissimus (Supplementary Video 1).

**Figure 3:**
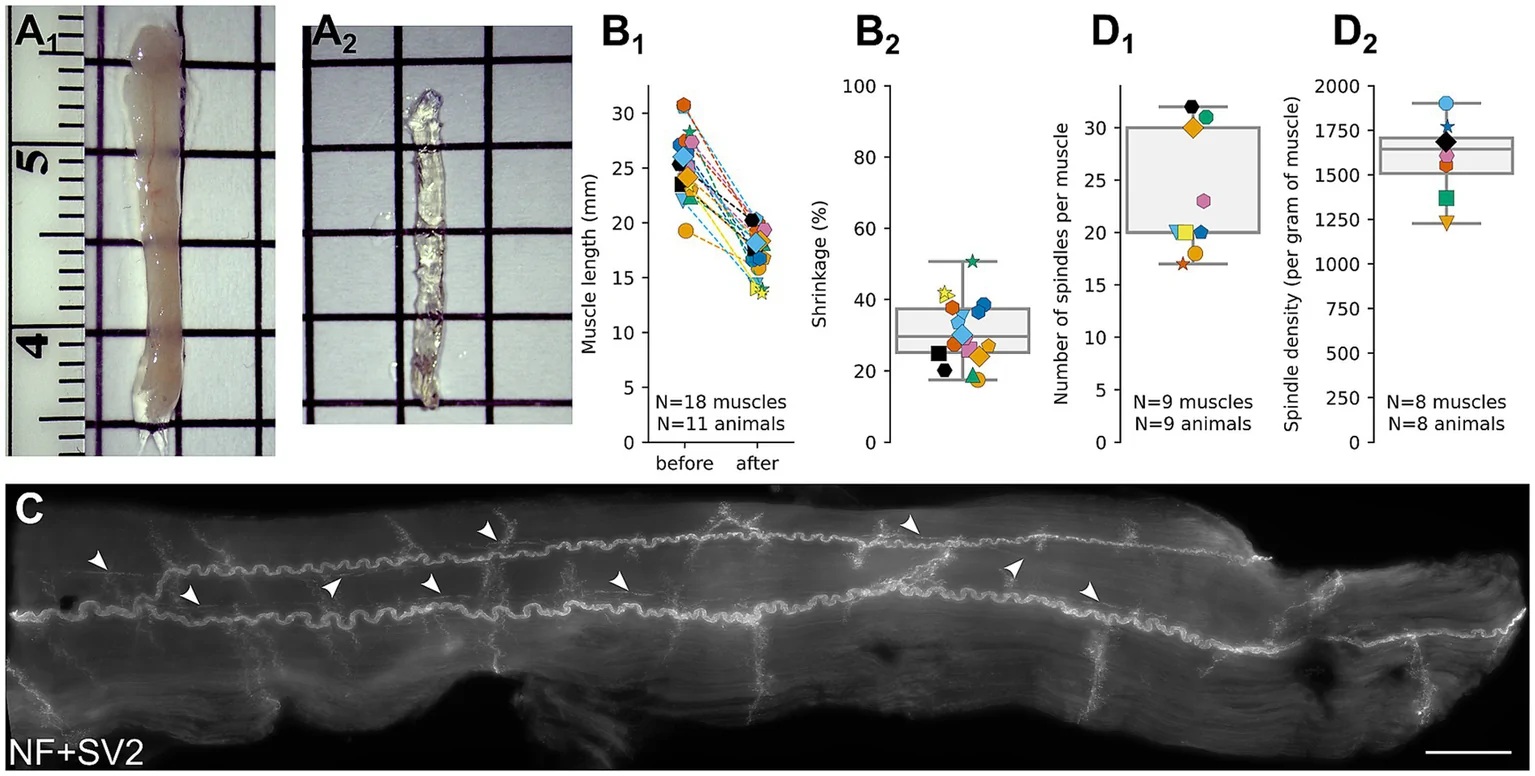
Optical clearing of neonatal rabbit tenuissimus muscles. **(A)** Pictures obtained before **(A**_**1**_**)** and after **(A**_**2**_**)** solvent-based optical clearing of a 12-day-old rabbit tenuissimus by FDISCO. In both images, each square is 5 × 5 mm. **(B)** Estimation of the shrinkage produced by the clearing process. **(B**_**1**_**)** Lengths of the muscles before and after clearing. **(B**_**2**_**)** Summary of the shrinkage amount. **(C)** Maximal-intensity projection of a tiled (8 × 3 tiles) z-stack (step size 20 μm, seven steps) epifluorescence image of a 8 day-old rabbit tenuissimus that was cut in half prior to optical clearing. In this half-muscle, at least nine spindles are readily visible (arrowheads), running parallel to the main nerve branch that serpentines through the whole length of the muscle. 10×/0.4 NA objective. Scale bar: 500 μm. **(D**_**1**_**)** Summary of the total number of spindles in each tenuissimus muscle and **(D**_**2**_**)** Summary of the spindle density. Each symbol represents a different animal. The boxplots show the three quartile values of the distribution. The “whiskers” extend to points that lie within 1.5 x inter-quartile distance of the lower and upper quartile. NF, neurofilament; SV2, synaptic vesicle glycoprotein 2.

Our optical clearing protocol was highly reproducible and resulted in robust tissue transparency with moderate shrinkage. The success rate of our technique for three-plex fluorescent labeling of motor end plates, spindle innervation, and outer capsule cells was 100% (*N* = 12 of 12 muscles from 7 rabbits, from two independent clearing preparations). We found that solvent-based optical clearing led to 31.1% ± 8.9% shrinkage of the tenuissimus, estimated from muscle length (*N* = 18 muscles isolated from 11 rabbits, from four independent clearing preparations) (Figures 3A,B). We observed a high density of spindles near the midline of the optically cleared muscle, parallel to the central neurovascular bundle (Figure 3C). We counted the number of spindles per tenuissimus using neurofilament, synaptic vesicle glycoprotein 2, and/or GLUT1 immunofluorescence as spindle markers (Figure 3D). On average, the tenuissimus contained 23.4 ± 5.9 spindles (*N* = 9 muscles isolated from 9 rabbits, from three independent clearing preparations). The average spindle density of the tenuissimus was 1,600 ± 217 spindles/g (*N* = 8 spindles, from 8 muscles isolated from 8 rabbits in 2 independent clearing preparations). To show that we could image spindles deep inside the muscle, we estimated the signal-to-noise ratio as a function of tissue depth using both confocal and lightsheet microscopy. As shown in Supplementary Figure 2, the intensity of the background fluorescence was roughly constant as a function of depth, and the intensity of the signal was strong enough to be clearly visualized and quantified.

**Figure 4:**
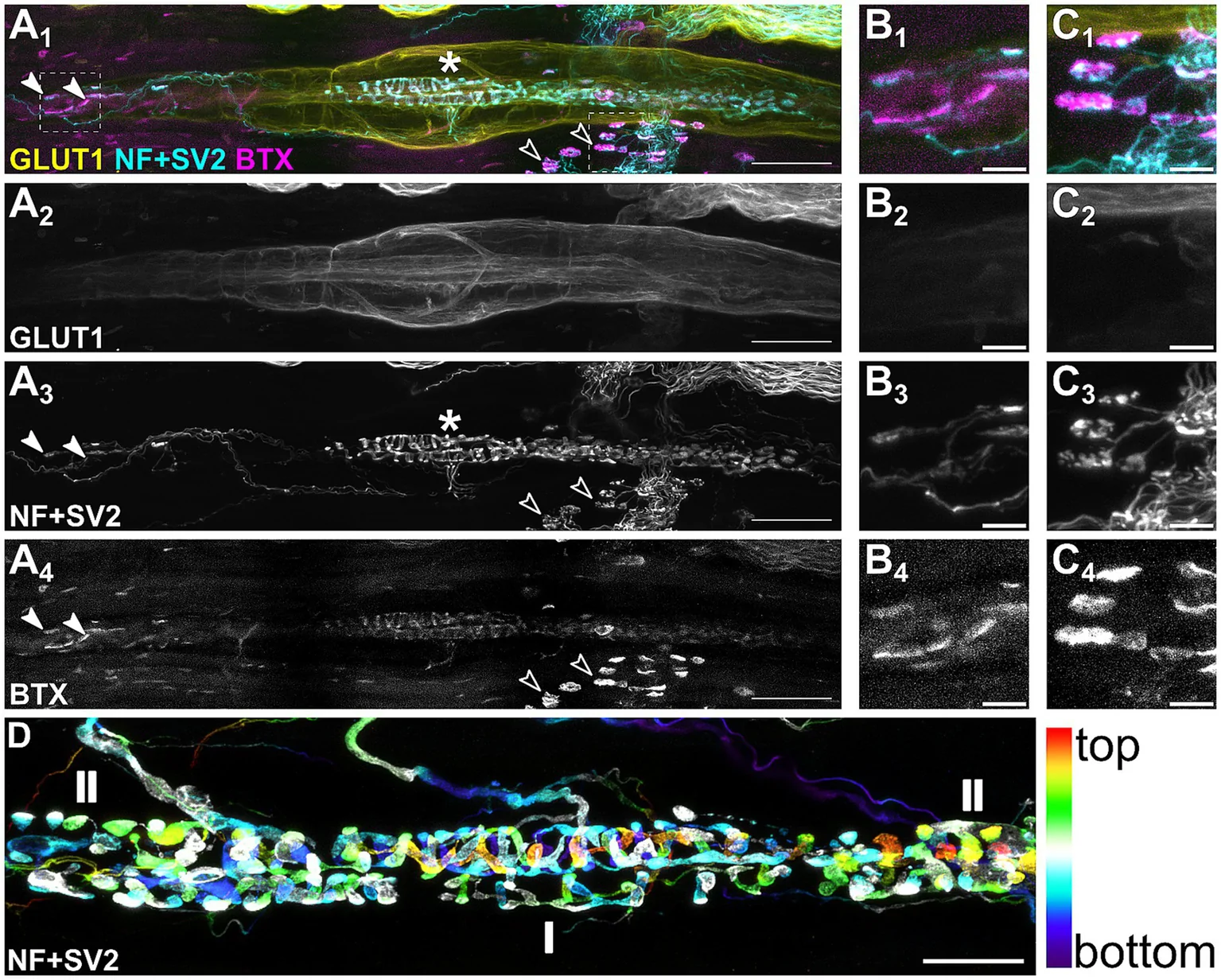
Representative images obtained from optically cleared triple-labeled tenuissimus muscles. **(A)** Maximum-intensity projection of a tiled z-stack confocal image of a single spindle from a P8 rabbit, showing the overlay **(A**_**1**_**)** of the GLUT1 labeling **(A**_**2**_**)**, pre-synaptic element labeling (neurofilament and SV2, **A**_**3**_) and the labeled post-synaptic receptors (α-BTX, **A**_**4**_). The asterisk * points to the equatorial region of the spindle. Empty arrowheads point to extrafusal α neuromuscular junctions, while filled arrowheads point to γ motor end plates. Scale bar: 50 μm. Tiled image acquired at 40×/0.6NA, pinhole size: 40 μm. **(B)** Zoom over the γ-fusimotor neuromuscular junctions circled with a dashed line in A. Same organization as in A, scale bar: 10 μm. **(C)** Zoom over the extrafusal α neuromuscular junctions circled with a dashed line in A. Same organization as in A, scale bar: 10 μm. **(D)** Depth-coded projection of a z-stack (0.6 μm step size, 33 steps) of the equatorial region of a spindle acquired with a 60×/1.4NA oil immersion objective. The color bar on the right shows how each color corresponds to a particular depth in the z-stack. The regions innervated by the primary and secondary endings are noted with I and II, respectively. Scale bar: 50 μm. BTX, bungarotoxin; GLUT1, glucose transporter 1; NF, neurofilament; SV2, synaptic vesicle glycoprotein 2.

Using our workflow, we were able to visualize spindle structures in their entirety (Figure 4). Spindle capsules were marked by immunofluorescent labeling of GLUT1, which was expressed by outer capsule cells (Figure 4A_2_). Interestingly, we observed that anti-rabbit immunoglobulin G (IgG) secondary antibody partly contributed to fluorescent labeling of the capsule structure even when rabbit anti-GLUT1 primary antibody was omitted (Supplementary Figure 1A). The spindle capsule is a specialized extension of the peripheral nerve perineurium and has diffusion barrier properties like the blood-nerve barrier (Ovalle and Dow, 1985). Since IgG localizes to the perineurium (Seitz et al., 1985), we suspect that anti-rabbit IgG secondary antibody labels endogenous rabbit IgG in the spindle capsule. Unfortunately, a commercial rabbit-to-rabbit blocking reagent (ScyTek Laboratories, Inc., catalog #RTR015, lot # 91203) was unsuccessful in eliminating this non-specific binding. However, the labeling obtained with the rabbit anti-GLUT1 and/or the anti-rabbit IgG was highly specific to the perineurium and was therefore adequate to visualize the spindle capsule. The equatorial region of the spindles was easily identifiable by neurofilament and synaptic vesicle glycoprotein 2 immunofluorescent labeling of the annulospiral endings (Figure 4A_3_). We attempted to label γ end plates by immunofluorescent labeling of acetylcholine receptor nicotinic alpha 1 subunit (Chrna1), but found that rat anti-Chrna1 antibody (DSHB, cat. #mAb35, lot # 12/5/24) was incompatible with our workflow (data not shown). Instead, spindle poles were identified by labeling γ-neuromuscular junctions with fluorescent α-BTX (filled arrowheads in Figure 4A). Although other optical clearing methods (Milgroom and Ralston, 2016; Williams et al., 2019; Zhu et al., 2026) were unsuccessful in labeling neuromuscular junctions, we found fluorescent labeling of motor end plates was uniformly retained when fluorescent α-BTX was applied to the muscle prior to immersion fixation. This signal was highly specific for both α and γ-neuromuscular junctions and starkly contrasted the control condition in which α-BTX was not applied (Supplementary Figures 1B–D). Intrafusal end plates were clearly distinguishable from extrafusal neuromuscular junctions. γ-neuromuscular junctions were isolated and found in relatively close proximity to the equatorial annulospiral endings (Figure 4B). On the other hand, α-neuromuscular junctions tended to be organised in grapes perpendicular to the main nerve branch (Figure 4C). Extrafusal end plates also tended to be much brighter and more complex than intrafusal end plates (Banks et al., 1985; Kucera and Walro, 1986; Arbuthnott et al., 1992). Based on these morphological differences, we were able to identify and study both α and γ neuromuscular junctions (Figures 4B–C).

**Figure 5:**
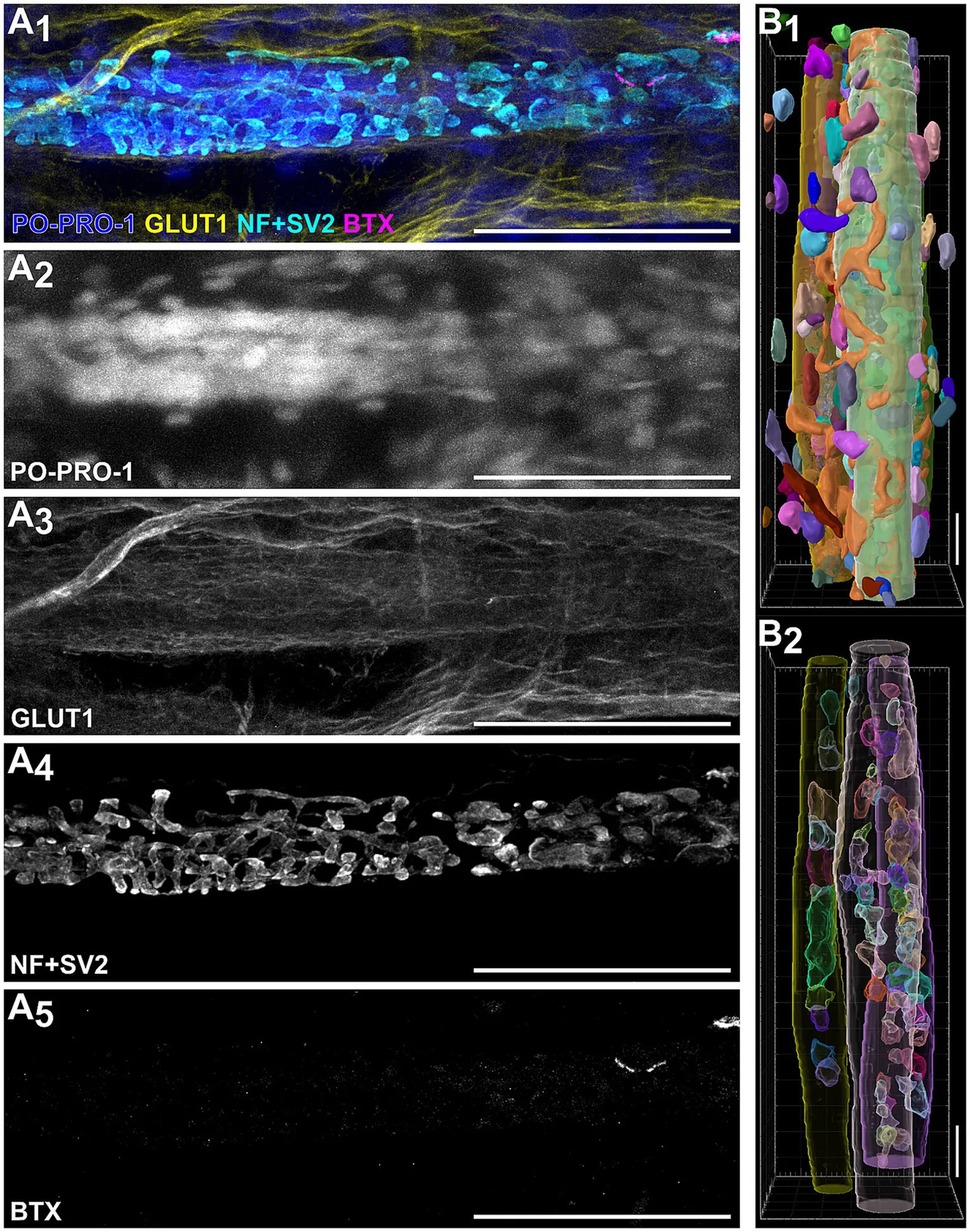
Quad-plex labeling of an optically cleared muscle spindle. **(A)** Maximum-intensity projection of a z-stack confocal image of a single spindle from a P5 rabbit, showing the overlay **(A**_**1**_**)** of the nuclear stain (PO-PRO-1, **A**_**2**_), GLUT1 labeling **(A**_**3**_**)**, pre-synaptic element labeling (neurofilament and SV2, **A**_**4**_) and the labeled post-synaptic receptors (α-BTX, **A**_**5**_). Scale bar: 50 μm. Image acquired with a 60×/1.4NA oil immersion objective, pinhole size 30 μm, z-stack acquired with a step size 0.3 μm, 87 steps. **(B)** Segmentation of the equatorial region of the same spindle. **(B**_**1**_**)** Shows a volumetric view of the segmented intrafusal muscle fibers, annulospiral endings and nuclei. **(B**_**2**_**)** The annulospiral ending and the nuclei outside the fibers were masked in order to visualize the nuclei that are inside each of the three fibers of this spindle, revealing the leftmost fiber to be a chain fiber, while the two fibers that are on top of each other on the right are bag fibers. Scale bars: 10 μm. Segmentation and analysis performed with Arivis v.4.5.0. BTX, bungarotoxin; GLUT1, glucose transporter 1; NF, neurofilament; SV2, synaptic vesicle glycoprotein 2.

To test compatibility with four-plex fluorescent staining, in one independent clearing preparation nuclei were counterstained with the monomeric cyanide nucleic acid stain PO-PRO-1 Iodide. Our workflow was amenable to conventional four-plex fluorescence microscopy (Figure 5); we were able to use the fourth channel to additionally visualize the nuclei, including the myonuclei of intrafusal fibers (Figure 5A). Nuclear counterstaining enabled us to visualize nuclear bag and chain fibers at the spindle equator (Figure 5B). Their difference in equatorial diameter was not very apparent, likely due to the relative immaturity of the sensorimotor system in the early postnatal period; it has been shown that the average cross-sectional area of bag fiber nuclei increases by 25 % between the first postnatal week and adulthood (Marchand and Eldred, 1969). Taken together, our protocol effectively combined fluorescent labeling of myonuclei, motor end plates, spindle sensory and motor innervation, and outer capsule cells for robust visualization of the complete muscle spindle.

### Our workflow enabled morphological analyses of muscle spindles

**Figure 6:**
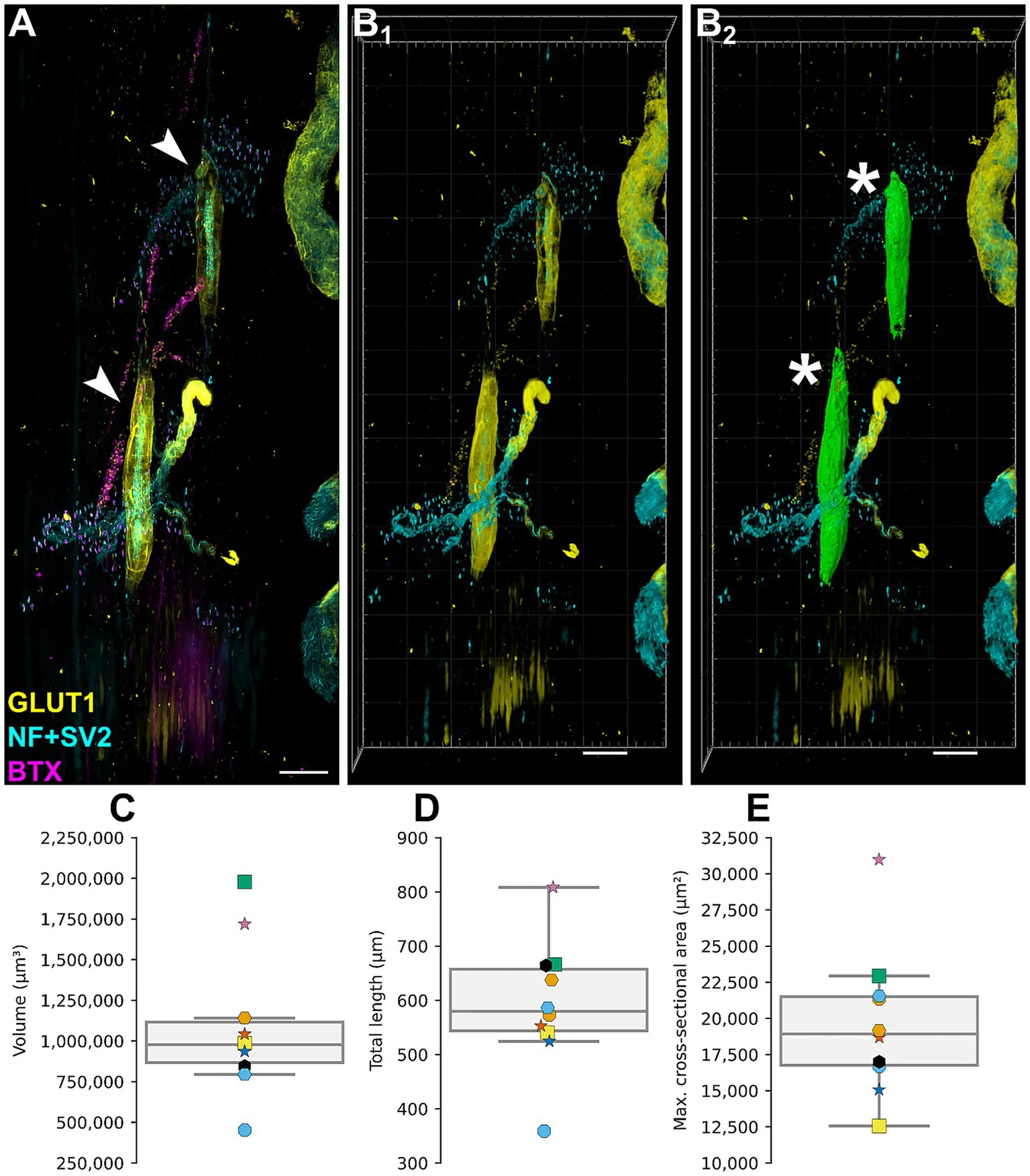
Morphological analysis of the optically cleared spindle capsule. **(A)** Maximum-intensity projection of a tiled z-stack lightsheet image showing two spindles (filled arrowheads) from a P8 rabbit. Zeiss Lightsheet 7, objective 20×/1.0NA. Scale bar: 100 μm. **(B)** Volumetric representation of the same image without **(B**_**1**_**)** and with **(B**_**2**_**)** the segmented capsules (green, asterisks). Scale bars: 100 μm. **(C–E)** Summary statistics of the total volume segmented **(C)**, the length of the capsule (as the maximum Feret diameter, **D**), and the maximum cross-sectional area in the XZ plane **(E)**. Each symbol represents a different rabbit. The boxplots show the three quartile values of the distribution. The “whiskers” extend to points that lie within 1.5 x inter-quartile distance of the lower and upper quartile. BTX, bungarotoxin; GLUT1, glucose transporter 1; Max., maximum; NF, neurofilament; SV2, synaptic vesicle glycoprotein 2.

Optical clearing of the intact muscle gave an unprecedented access to the three-dimensional structure of the annulospiral endings of the Ia afferent fibers around the equatorial region of the intrafusal fibers. Z-stacks obtained on a confocal microscope highlighted the complex organization of these structures (Figure 4A_3_) and allowed us to evaluate their morphology. We counted the number of intrafusal fibers, which could be identified by the spirals of the primary afferents and faint GLUT1 labeling around the fibers. We found that the average number of intrafusal fibers per muscle spindle was 3.2 ± 0.4 fibers (*N* = 9 spindles, from 8 muscles isolated from 8 rabbits, in three independent clearing preparations). Although confocal imaging provides the highest level of detail, it is limited by the working distance of the objectives, which is sometimes too short to focus on the spindles deep inside the muscle, particularly in older animals. Lightsheet microscopy was used to produce high resolution volumetric images of the muscle (Supplementary Video 1), including muscle spindles and their capsules (Figure 6). GLUT1 immunofluorescence was used to segment spindle capsules in lightsheet photomicrographs. The total spindle volume was about 0.001 mm^3^ (1,086,415 ± 446,325 μm^3^ (*N* = 10 spindles, from 5 muscles isolated from 5 rabbits, in one independent clearing preparation) but varied substantially from spindle to spindle (Figure 6C). The total length of the spindle capsule was 591 ± 117 μm (*N* = 10 spindles, from 5 muscles isolated from 5 rabbits, in one independent clearing preparation; Figure 6D), and the maximum cross section area of the capsule (in the plan perpendicular to its major axis) was 19,597 ± 5,099 μm^2^ (*N* = 10 spindles, from 5 muscles isolated from 5 rabbits, in one independent clearing preparation; Figure 6E).

Overall, we show here that the combination of optical clearing and confocal or lightsheet microscopy (especially for thicker samples) allows morphological analysis of the three-dimensional structure of muscle spindles.

## Discussion

### Advantage of our protocol

Morphometric analysis of muscle spindles typically involves labor-intensive teasing of silver-impregnated muscle spindles, longitudinal muscle cryosectioning, or serial transverse cryosectioning with three-dimensional reconstruction (Kim et al., 2007; Muller et al., 2008; Gerwin et al., 2020; Gartych et al., 2021; Piotr et al., 2023; Kawai-Takaishi and Hosoyama, 2025). Our study demonstrates a reproducible workflow that overcomes major limitations of conventional spindle histology and provides a powerful platform for investigating muscle spindle development, plasticity, aging, and pathology *in situ*, in intact muscles. Our workflow was reproducible, yielded high signal-to-noise ratios (across large z-depths) for multi-channel fluorescence microscopy, and enabled volume imaging of the entire spindle structure, including its capsule and its sensory and fusimotor innervation (Figure 4). A major advantage of our technique was its suitability for standard four-channel fluorescence microscopy (Figure 5). We quantitatively validated our protocol, performing spindle morphometry in the neonatal rabbit tenuissimus; we counted intrafusal fibers and measured spindle capsule volume, length, and maximum cross-sectional area from segmented immunofluorescent photomicrographs (Figure 6). Others have used transgenic fluorescent reporter lines like Thy1-eGFP to visualize spindle annulospiral endings in mouse skeletal muscle optically cleared with hydrophilic-based [CUBIC (Shi et al., 2025) and SeeDB (Nikolaou et al., 2015)] and hydrogel-based [PACT (Bornstein et al., 2023)] methods, but none have labeled spindle poles (γ motor end plates) or used immunofluorescence to label spindle innervation. The protocol developed in this study may be useful for other groups studying spindle structure and/or those more broadly interested in visualizing neuromuscular junctions, particularly in species where no transgenic reporter lines exist.

Fluorescent dye-conjugated-α-BTX binds to the α subunit of nicotinic acetylcholine receptors and is the gold standard reagent for labeling motor end plates at α- and γ-neuromuscular junctions in skeletal muscle (Anderson and Cohen, 1974). While widely used in standard immunofluorescence experiments, fluorescent dye-conjugated α-BTX is challenging to pair with optical clearing of skeletal muscle. This reagent is incompatible with hydrogel-based optical clearing methods when the key component sodium dodecyl sulfate (SDS) is used for delipidation (Milgroom and Ralston, 2016; Williams et al., 2019); exclusion of SDS in the MYOCLEAR hydrogel-based method yields strong α-BTX labeling, but causes weaker antibody penetration and high blue-to-red range autofluorescence which limits co-labeling immunofluorescence experiments to those involving spectral unmixing of far red light (Williams et al., 2019). Fluorescent α-BTX has never been successfully paired with iDISCO (Renier et al., 2014; Zhu et al., 2026), and has only been shown to reliably label motor end plates in rodent skeletal muscle optically cleared using other solvent-based protocols when administered by intravenous administration (Chen et al., 2016; Qi et al., 2019; Yin et al., 2019). The intravenous approach for motor end plate labeling is complex, since tail vein administration of fluorescent α-BTX requires a dosage (0.3 μg/g) above the LD50 for the mouse (0.113 μg/g) and for staining efficacy requires a 1–2 h delay under anesthesia before euthanasia and transcardial perfusion with paraformaldehyde. To our knowledge, ours is the first demonstration that fluorescent α-BTX can be chemically fixed at nicotinic acetylcholine receptors by initial immersion followed by fixation in paraformaldehyde and that this fluorescent labeling of motor end plates is compatible with iDISCO and solvent-based optical clearing. This approach may be useful for those interested in studying neuromuscular junction biology (γ and/or α) in optically cleared skeletal muscle.

### The tenuissimus muscle

Several decades ago the tenuissimus muscle was the subject of foundational research on muscle spindle physiology and fusimotor innervation, due to its high spindle density (Hunt and Kuffler, 1951, p. 1951; Boyd and Ward, 1975; Emonet-Dénand et al., 1975; Barker et al., 1976; Banks, 1991; Celichowski et al., 1994). The tenuissimus generates very small forces because it is a thin muscle with few extrafusal fibers (Lev-Tov et al., 1988). It contains many spindles aligned in series near the midline of the muscle, parallel to the central neurovascular bundle (Figure 3C)(Miyoshi et al., 1979; Patten and Ovalle, 1992). This anatomical feature of the tenuissimus and its small size support the idea posed by (Patten and Ovalle, 1992) that the high degree of proprioceptive feedback provided by this muscle fine tunes gross movement in the neighboring biceps femoris muscle, which has a much larger force generative capacity (Peck et al., 1984; Patten and Ovalle, 1992). In this study, we adapted contemporary immunofluorescence and optical clearing methods for three-dimensional visualization of tenuissimus muscle spindles *in situ*. Our work returns attention to the tenuissimus and highlights its unique suitability for investigations in muscle spindle biology.

Other muscles are amenable to whole-mount microscopy, like the extensor digitorum longus (EDL) as done by (Vaughan et al., 2015), which decreases labor intensity and maximizes the number of spindles for morphometric analysis. More recently, (Bornstein et al., 2023) demonstrated the great utility of optical clearing for *in situ* visualization of muscle spindles in the EDL, which has a calculated spindle density of 1,026 spindles/gram of muscle in the adult mouse (Lian et al., 2022). In the current study, we show that the rabbit tenuissimus had an average of 23 spindles per muscle and a very high spindle density of ~1,600 spindles per gram. This count is higher than reported for the cat (~15 spindles; Boyd and Davey, 1968) and hamster tenuissimus (~18 spindles; Patten and Ovalle, 1992). The high spindle density of the rabbit tenuissimus makes it particularly well-suited for spindle morphological analysis.

### Choice of the neonatal rabbit model

We applied our protocol to neonatal rabbit muscle because of its translational research potential in the area of neuromuscular development. Compared to rodents vs. humans, phases of central nervous development are more similar in rabbits vs. humans (Semple et al., 2013; de Almeida da Anunciação et al., 2021), likely reflecting their closer phylogenetic relationship (Graur et al., 1996). Also, comparative anatomy highlights a greater similarity in muscle structure between humans and rabbits vs. humans and mice (Paul, 2001). Muscle spindle morphology is dynamic, and structural properties change with age; we analyzed a time point when the neuromuscular system is immature. The sensory nerve terminal transitions from a web-like to a spiral appearance during early postnatal spindle development in rodents (Maeda et al., 1985; Osawa et al., 1988; Kröger and Watkins, 2021), and in aged mice, sensory axon blebbing, Ia afferent atrophy at muscle spindle entry, and reduced spirality of annulospiral endings has been reported (Kim et al., 2007; Kawai-Takaishi and Hosoyama, 2025). While our pipeline is directly applicable to studies of proprioception and/or reflexive control of muscle tone in developing rabbits, and also to rabbit models of cerebral palsy (Derrick et al., 2004; Georgiadis et al., 2008; Saadani-Makki et al., 2008; Drobyshevsky et al., 2015), the same approach should be readily adaptable to other muscles, particularly rodent skeletal muscles that are comparable in size or smaller than the neonatal rabbit tenuissimus.

### Limitations

As with most solvent-based optical clearing protocols, tissue shrinkage was observed and is unavoidable. However, because shrinkage is expected to occur consistently across samples processed using the same pipeline, it does not preclude relative comparisons between experimental groups when uniform processing conditions are maintained.

## Conclusion

In this study, we present a novel workflow for fluorescent labeling and volume imaging of muscle spindles in the neonatal rabbit tenuissimus, a whole mount-amenable muscle that is remarkably well-suited for spindle morphometry due to its high spindle density. Our novel approach enables in situ investigations into muscle spindle structure, including the capsule, annulospiral endings and intrafusal neuromuscular junctions in the contexts of health and disease.

## Data Availability

The raw data supporting the conclusions of this article will be made available by the authors, without undue reservation.

## Supplementary material

SUPPLEMENTARY FIGURE 1 Characterizations of background fluorescence. **(A)** Maximum-intensity projection of a tiled z-stack confocal image of a single spindle from a P8 rabbit that was labeled with fluorescent secondary antibodies only (Alexa Fluor 488 Goat Anti-Rabbit IgG, and Alexa Fluor 555 Goat anti-Mouse IgG). Primary antibodies anti-GLUT1, anti-NF, and anti-SV2 were omitted. Scale bar: 50 μm. Objective 40×/0.6NA. Pinhole size 60 μm, step size 1.2 μm, 38 steps. Acquisition was performed with similar laser powers and gains as Figure 4. **(B)** Maximum-intensity projection of a tiled z-stack confocal image of a single spindle from a P5 rabbit that was labeled with antibodies against GLUT1 and NF + SV2 and their corresponding fluorescent secondary antibodies, but without pre-treatment with α-bungarotoxin-647. Scale bar: 50 μm. Objective 40×/0.6NA. Pinhole size 60 μm, step size 1.2 μm, 34 steps. **(C)** Zoom on the extrafusal α neuromuscular junctions circled with a dashed line in **B**. Scale bar: 10 μm. **(D)** Zoom on the intrafusal γ neuromuscular junctions circled with a dashed line in **B**. Scale bar: 10 μm. Acquisition was performed with similar laser powers and gains as Figure 4. GLUT1, glucose transporter 1; IgG, Immunoglobulin G; P, postnatal day; NA, numerical aperture; NF, neurofilament; SV2, synaptic vesicle glycoprotein 2.

SUPPLEMENTARY FIGURE 2 Estimation of signal-to-noise ratios in cleared samples. **(A–D)** Estimation of signal-to-noise ratios in confocal images. **(A)** Maximum intensity projection in the z direction of a z-stack through the whole thickness of a sample from a P8 rabbit. Objective 40×/0.6NA. Pinhole size 60 μm. Step size 10 μm, 20 steps. **(B**_**1**_**)** Maximum intensity projection of the GLUT1 channel in the XZ plane, showing that the background intensity is relatively homogenous throughout the thickness of the sample. **(B**_**2**_**)** Plot of the intensity of the background and signal intensities as a function of depth for the GLUT1 channel. The background signal is estimated from the median fluorescence intensity in the channel at each z-plane, while the signal is estimated from the maximum fluorescence intensity in the channel at each z-plane. **(C)** Same as B for the NF + SV2 channel. **(D)** Same as B for the BTX channel. Scale bars: 50 μm. **(E–H)** Estimation of signal-to-noise ratio in lightsheet images (Zeiss Lightsheet 7, detection objective 20×/1.0NA, light sheet thickness 3.91 μm, step size 5 μm, 82 steps). Same organization as in **A–D**. Scale bars: 50 μm. **(I)** Plot of the intensity of the background and signal intensities as a function of depth for the GLUT1 **(I**_**1**_**)**, NF + SV2 **(I**_**2**_**)**, and BTX **(I**_**3**_**)** channels averaged over all samples (*N* = 8 muscles isolated from 6 animals, from one clearing experiment, imaged by lightsheet microscopy). The thick lines represent the mean value, while the shaded areas represent the 95% confidence interval. BTX, bungarotoxin; GLUT1, glucose transporter 1; NF, neurofilament; SV2, synaptic vesicle glycoprotein 2.

SUPPLEMENTARY VIDEO 1 Volumetric 3D reconstruction of part of a tenuissimus muscle from a P8 rabbit, imaged using a MegaSPIM lightsheet microscope (detection objective 22×/0.7NA). Fluorescence from the NF + SV2 immunolabeling. P, postnatal day; NA, numerical aperture; NF, neurofilament; SV2, synaptic vesicle glycoprotein 2.
